# Prognostic value of posttreatment ^18^F-FDG PET/CT and predictors of metabolic response to therapy in patients with locally advanced cervical cancer treated with concomitant chemoradiation therapy: an analysis of intensity- and volume-based PET parameters

**DOI:** 10.1007/s00259-018-4077-1

**Published:** 2018-08-02

**Authors:** Giacomo Maria Lima, Antonella Matti, Giulio Vara, Giulia Dondi, Nicoletta Naselli, Eugenia Maria De Crescenzo, Alessio Giuseppe Morganti, Anna Myriam Perrone, Pierandrea De Iaco, Cristina Nanni, Stefano Fanti

**Affiliations:** 1 Nuclear Medicine Department, S.Orsola-Malpighi Hospital, University of Bologna, Bologna, Italy; 2 Oncologic Gynecology Unit, S.Orsola-Malpighi Hospital, University of Bologna, Bologna, Italy; 3 Radiology Unit, Department of Diagnostic and Preventive Medicine, S.Orsola-Malpighi Hospital, University of Bologna, Bologna, Italy; 4 Radiation Oncology Centre, Department of Experimental, Diagnostic and Specialty Medicine, S.Orsola-Malpighi Hospital, University of Bologna, Bologna, Italy

**Keywords:** Cervical cancer, LACC, FDG PET/CT, Prognostic value, Chemoradiation therapy

## Abstract

**Purpose:**

To investigate the prognostic value of posttreatment ^18^F-FDG PET/CT in patients with locally advanced cervical cancer (LACC) treated with concomitant chemoradiation therapy (CCRT). The secondary aim was to assess the possible role of intensity-based and volume-based PET parameters including SUVmax, SUVmean, MTV and TLG, and clinical parameters including age, pathology, FIGO stage and nodal involvement as factors predicting response to treatment.

**Methods:**

This retrospective study included 82 patients affected by LACC treated with CCRT. All patients underwent ^18^F-FDG PET/CT both before and after treatment. The posttreatment PET/CT scans were used to classify patients as complete metabolic responders (CMR) or non-complete metabolic responders (N-CMR) according to the EORTC criteria. Kaplan-Meier analysis was used to evaluate differences in overall survival (OS) between the CMR and N-CMR groups. Student’s *t* test, Pearson’s chi-squared test and logistic regression were used to investigate the possible value of PET and clinical parameters as predictors of metabolic response to therapy.

**Results:**

Kaplan­Meier analysis showed a highly significant difference in OS between the CMR and N-CMR groups (log-rank test *p* < 0.0001). Significant independent predictors of response to therapy were MTV (*p* = 0.019, odds ratio = 1.015, 95% CI = 1.002–1.028, Nagelkerke *R*^2^ = 0.110), TLG (*p* = 0.045, odds ratio = 1.001, 95% CI = 1.000–1.002, Nagelkerke *R*^2^ = 0.081) and nodal involvement (*p* = 0.088, odds ratio = 2.361, 95% CI = 0.879–6.343, Nagelkerke *R*^2^ = 0.051).

**Conclusion:**

^18^F-FDG PET/CT-based response assessment using the EORTC criteria reliably predicts OS in LACC patients treated with CCRT. In our cohort of patients, pretreatment MTV and TLG and nodal involvement were predictors of response to therapy. MTV was the best predictor of response. However, its additional risk value seems to be low (MTV odds ratio = 1.015).

## Introduction

### Background

Cervical cancer is the fourth most common cancer in women with an estimated 528,000 new cases reported in 2012. A major part (around 85% of cases) of the global burden occurs in developing countries [1, 2]. This is due to a lack of screening that is the most important public health intervention to reduce both the incidence and mortality of the disease [3–6]. Cervical tumours are staged using the International Federation of Gynecology and Obstetrics (FIGO) classification [7]. Surgery is considered only in patients with earlier stages of cervical cancer (up to FIGO stage IIa) without risk factors. Otherwise the standard of care for locally advanced cervical cancer (LACC; FIGO Ib2–IVa) is concomitant chemoradiation therapy (CCRT) [8].

^18^F-FDG PET/CT has a well-established role in the management of patients with cervical cancer, especially in staging, radiotherapy planning, response assessment and recurrence of LACC as stated in American National Comprehensive Cancer Network (NCCN) guidelines [9–11].

### Detection, staging and radiation therapy planning

Clinical suspicion of cervical cancer is confirmed by pathology findings, and imaging examinations are routinely used for locoregional and distance staging and follow-up [8]. Pelvic MRI imaging is the best tool to assess locoregional disease. It can evaluate tumour size, stromal penetration and parametrial invasion [12, 13] and the potential involvement of the bladder, rectum and pelvic sidewall [14]. CT and PET/CT imaging are used for whole-body staging [13]. PET/CT has a limited role in local primary staging [15]. The most important role is in the detection of distant metastasis and pathological lymph nodes with better performance than CT, especially for nonpathological enlarged lymph node metastases [16, 17]. In patients with LACC, PET/CT is often used to plan the correct radiation therapy field. PET/CT has been shown to influence external-beam radiotherapy planning by allowing modification of the treatment field and customization of the radiation dose [18–21].

### Treatment response

Depending on stage, cervical carcinoma is treated with surgery, radiation therapy or chemotherapy. Several studies have shown the value of PET/CT in the assessment of treatment response [22, 23]. With regard to chemoradiation therapy, the NCCN guidelines recommend that PET/CT be performed 3–6 months after the end of treatment. The key role of posttreatment PET/CT is the early detection of locoregional therapy failure that indicates the need for salvage therapy, and the identification of unsuspected asymptomatic distant metastasis. Radiotherapy-induced inflammatory changes can lead to false-positive ^18^F-FDG uptake, and thus the timing of the posttreatment PET/CT examination is crucial [24–26]. Persistent FDG uptake at the end of chemoradiotherapy is associated with a poor 5-year survival prognosis [22, 27]. The role of interim PET performed during radiation therapy is not yet unequivocally defined [28].

### Recurrent disease

The accurate localization of recurrent disease is critical and guides management and the selection of appropriate therapies. In these patients, earlier intervention leads to a better outcome [29]. In this setting, the role of PET/CT is to localize the recurrence so that patients can be properly referred to surgery (in those with pelvic recurrence) or salvage radiotherapy (in those with extrapelvic recurrence) [30–32].

### Aim

The aim of this study was to determine the prognostic value of posttreatment ^18^F-FDG PET/CT in patients with LACC treated with CCRT. For this purpose, the prognostic value of the criteria developed by the European Organization for Research and Treatment of Cancer (EORTC criteria) was evaluated with a survival analysis. We also assessed the possible role of pretreatment intensity-based and volume-based PET parameters and clinical data including maximum and mean standardized uptake values (SUVmax, SUVmean), metabolic tumour volume (MTV), total lesion glycolysis (TLG), age, pathology findings, FIGO stage and nodal involvement as factors predicting response to treatment.

## Materials and methods

### Population characteristics

The clinical records of all patients referred to our centre for cervical cancer from January 2008 to January 2018 were analysed. Among these patients, 82 affected by LACC and treated with CCRT were retrospectively enrolled. Other inclusion criteria were: proven diagnosis of cervical cancer, FIGO stage IIA–IVA (with or without positive paraaortic nodes), availability of full clinical history and follow-up data, CCRT to the pelvis (with or without an external beam boost to positive pelvic or para-aortic nodes together with a brachytherapy boost to the tumour as a single treatment option), and ^18^F-FDG PET/CT scans performed before and after treatment. Exclusion criteria were: previous surgical treatment of the cervical tumour, and FIGO stage IB2 (because the treatment is not unequivocally defined). After the end of therapy all patients were clinically evaluated by a gynaecologist (physical examination and ultrasonography or MRI where indicated). All patients gave permission to use their clinical data for scientific purposes.

### Radiopharmaceuticals and imaging protocol

Each patient underwent a ^18^F­FDG PET/CT for both staging and restaging at least 3 months after the end of treatment (range 3–12 months, mean 6.7 ± 3.0 months). All PET/CT scans were reviewed by two experienced nuclear medicine physicians.

Whole-body FDG PET/CT was performed using standard procedures. Briefly, 3 MBq/kg of ^18^F-FDG was intravenously injected. All patients were required to fast for 6 h. The uptake time was 60 min in all patients. Images were acquired on a 3-D tomograph (Discovery STE; GE) for 2 min per bed position. A low-dose CT scan (120 kV, 80 mA) was performed both for attenuation correction and to provide an anatomical map. PET images were reconstructed using an iterative 3-D ordered subsets expectation maximization method with two iterations and 20 subsets, followed by smoothing (with a 6-mm 3-D gaussian kernel) with CT-based attenuation, scatter and random coincidence event correction.

### Image analysis and interpretation criteria

For each scan, SUVmax, SUVmean, MTV and TLG were measured both in the primary cervical lesion and in nodal or distant lesions (when present).

MTV measurement was calculated on FDG PET/CT images using a semiquantitative (40% threshold) analysis, corrected with visual evaluation of the tracer uptake in the neoplastic lesions to avoid missing tumour at the boundaries. SUVmax and SUVmean normalized to body weight were calculated within the MTV defined as above. TLG values of the primary lesions were calculated as the product of MTV and SUVmean [33]. SUVmean, MTV and TLG were measured both in the primary lesion and in distant lesions. The final MTV value was the sum of the MTVs of every lesion and the final TLG value was the sum of the TLGs of every lesion. When the bladder (filled with radioactive urine even after voiding) was very close to the primary lesion, visual correction of the adjacent region of interest margin was necessary.

**Fig. 1:**
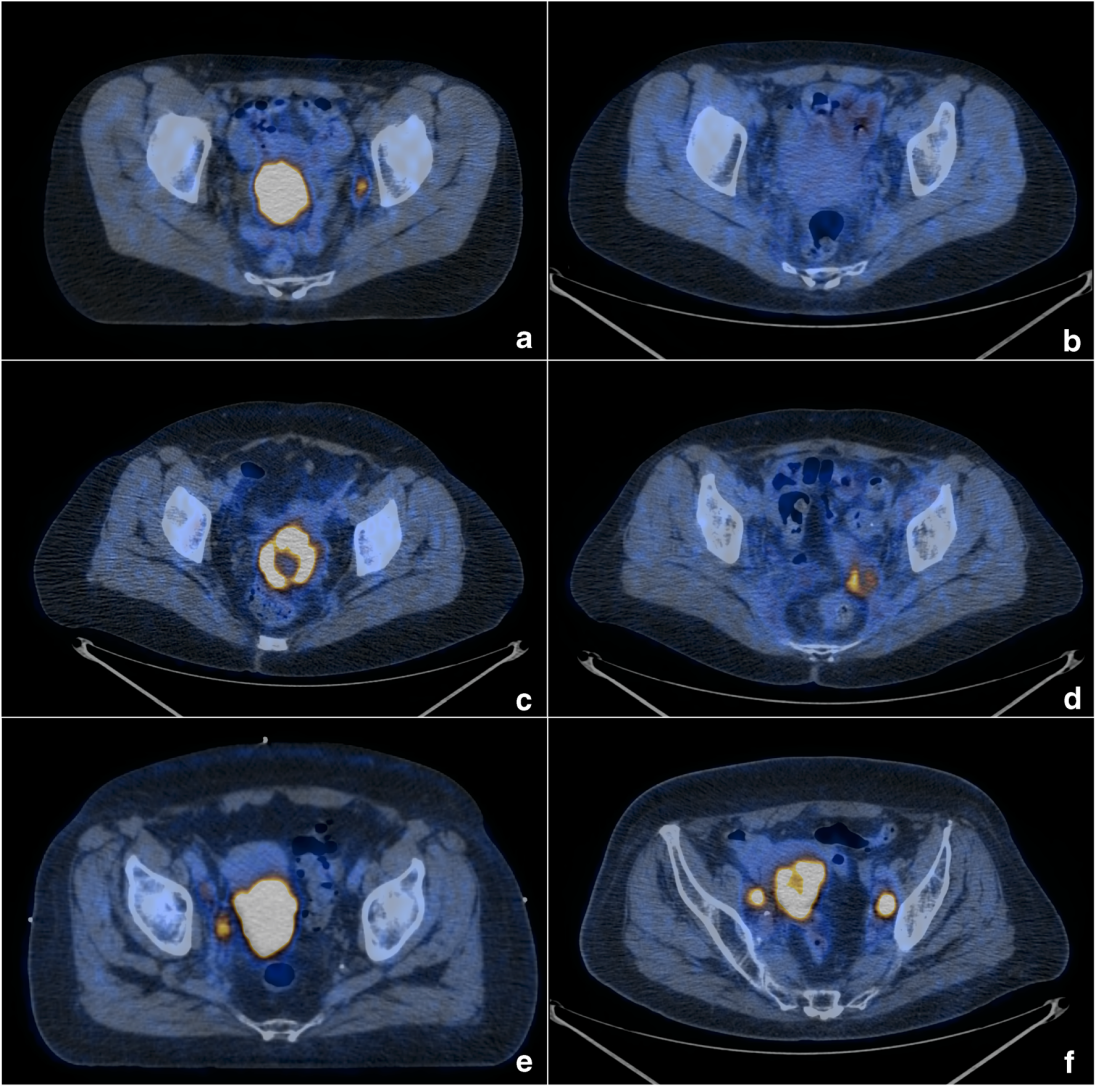
Axial PET/CT images before and after treatment: **a**, **b** showing a complete metabolic response; **c**, **d** showing a partial metabolic response; **e**, **f** showing progressive disease

The posttreatment PET/CT scans were used to classify patient responses into four categories according to EORTC criteria: complete metabolic response (CMR, no FDG uptake within the tumour volume), partial metabolic response (PMR, SUVmax reduction greater than 25% after treatment), stable metabolic disease (SMD, SUVmax increase or decrease less than 25%), progressive metabolic disease (PMD, SUVmax increase greater than 25% or increase in the extension of tumour uptake greater than 20% in the longest dimension or the appearance of new ^18^F-FDG uptake) [34]. Patients with PMR, SMD and PMD were then grouped as non-complete metabolic responders (N-CMR) (Fig. 1).

### Chemoradiotherapy

#### External beam radiotherapy

All patients underwent pelvic 3-D conformal external beam radiotherapy. The prescribed dose to the pelvis was 46 Gy in fractions of 2 Gy. The treatment volumes were delineated as follows. The clinical target volume (CTV) was defined as the gross tumour volume, uterus, parametria, upper third of the vagina, obturator, and presacral, iliac (common, internal and external) and paraaortic lymph nodes (if PET-positive) with an expansion of 7 mm. The CTV boost was defined as lymphadenopathies with high FDG uptake plus an expansion of 7 mm. The planning target volumes (PTV and PTV boost) were obtained by adding a 1-cm CTV to PTV isotropic expansion.

#### Chemotherapy

Cisplatin (40 mg/m^2^) was administered once a week during the period of external radiotherapy.

#### Brachytherapy

After radiochemotherapy all patients underwent a brachytherapy boost, with the high dose-rate or pulsed dose rate technique. The dose was prescribed according to the International Commission on Radiation Units (ICRU) Report 38 recommendations.

### Statistical analysis

All statistical analyses were performed using SPSS version 24 (IBM Corp., Armonk, NY). Kaplan-Meier analysis was used to evaluate differences in overall survival (OS) between the CMR and N-CMR groups and the log-rank test was used to evaluate the significance of differences between survival curves [35]. We also investigated whether SUVmax, SUVmean, MTV and TLG (measured on the pretreatment scan), age, pathology findings, FIGO stage and nodal involvement could predict the metabolic response to therapy. According to the data distribution, Student’s *t* test was used to compare the means of continuous variables, and Pearson’s chi-squared test was used to compare the frequencies of categorical variables. Univariate logistic regression was used to evaluate the associations between independent variables and the metabolic response to therapy (CMR vs. N-CMR, dependent variable) [36]. Independent variables are reported in terms of odds ratios (OR) and their 95% confidence intervals (CI). Significant independent variables (*p* < 0.1) were included in the multivariate backward logistic regression.

## Results

The main characteristics of the study population, including age, body mass index, pathology, FIGO stage, nodal involvement and follow-up, are presented in Table 1. Of the 82 patients, 23 died during the observation period. The timing of the posttherapy PET/CT scans did not differ significantly between the CMR group (6.9 ± 2.7 months) and the non-CMR group (6.5 ± 3.5 months).

**Table 1:** Characteristics of the 82 enrolled patients

Characteristic	Value
Clinical data, *n* (range)	
Age (years), mean (range)	61.3 (28–94)
BMI (kg/m^2^), mean (range)	25.4 (18–42)
Died during observation period, *n* (%)	23 (28)
FIGO stage, *n* (%)	
IIA	3 (3.7)
IIB	54 (65.8)
IIIA	4 (4.9)
IIIB	12 (14.6)
IVA	9 (11.0)
Nodal involvement, *n* (%)	
Nodal disease at staging	44 (53.6)
Lumboaortic nodal disease at staging	11 (13.4)
Pathology, *n* (%)	
Squamous	64 (78)
Adenocarcinoma	15 (18.3)
Other	3 (3.7)
Follow-up (months), mean (range)	50.8 (8.6–98.4)

According to EORTC criteria, of the 82 patients, 57 had CMR, 8 had PMR, 2 had SMD and 15 had PMD. The N-CMR group (those with PMR, SMD and PMD) included 25 patients. All patients were also clinically evaluated after chemoradiation treatment. Of the 57 patients in the CMR group, 48 were classified as clinical responders (84.2% concordance), and of the 25 patients in the N-CMR group, 10 were classified as clinical nonresponders (40% concordance). Even though clinical response is routinely assessed in LACC patients, in our study, the PET/CT scan led to reclassification of 24 of the 82 patients (29.3%).

### Survival analysis

**Fig. 2:**
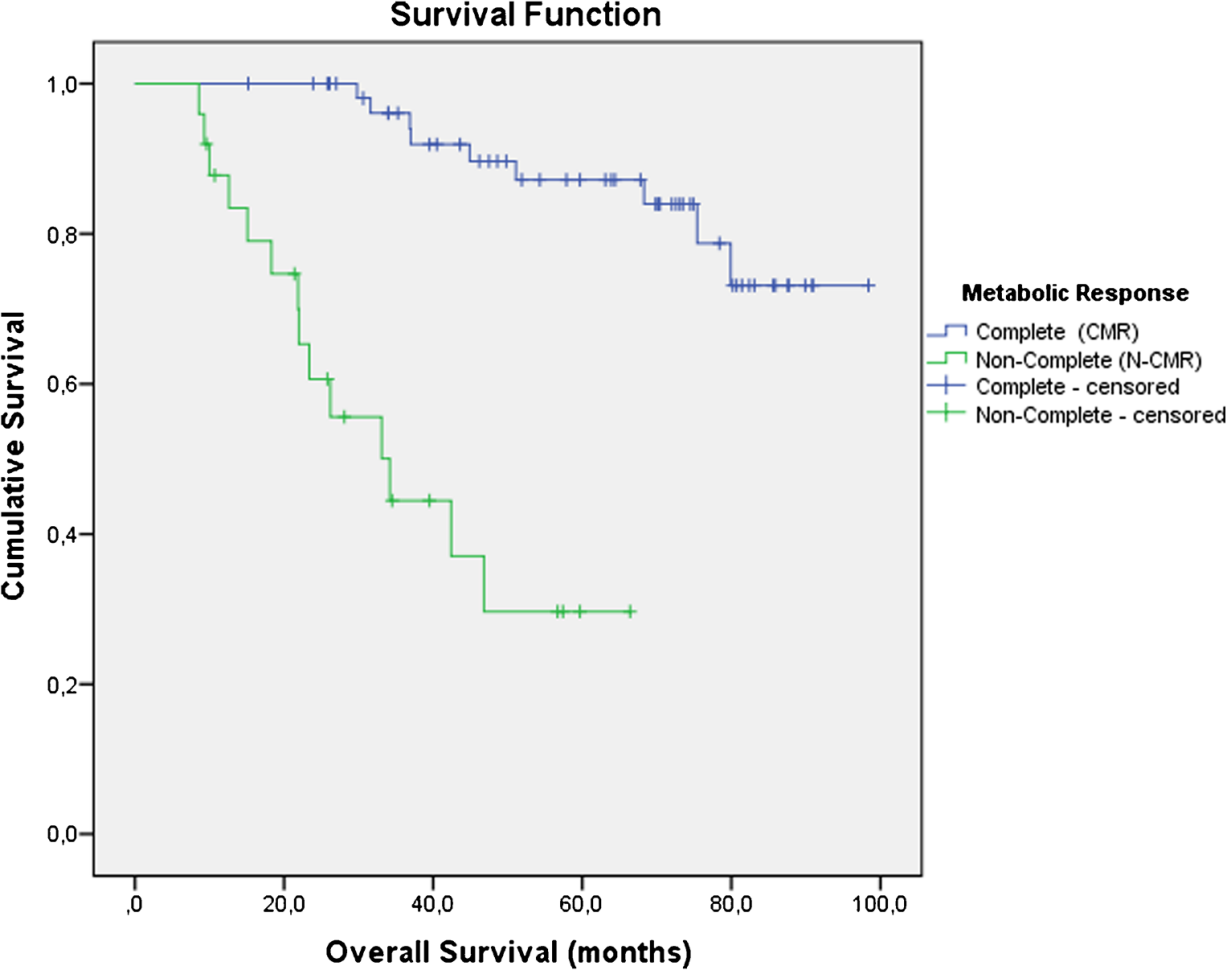
Kaplan­Meier survival curves. There was a highly significant difference in overall survival between the CMR and N-CMR groups (*p* < 0.0001)

Kaplan­Meier analysis showed a highly significant difference in OS between the CMR and N-CMR groups (log-rank test *p* < 0.0001; Fig. 2). Of the 57 patients in the CMR group and the 25 patients in the N-CMR group, 9 (15.78%) and 14 (56.00%), respectively, died during follow-up.

### Comparison of variables between CMR and N-CMR groups

Student’s *t* test and Pearson’s chi-squared test results are presented in Tables 2 and 3, respectively. The mean pretreatment MTV and TLG values were significantly different between the CMR and N-CMR groups (*p* = 0.008 and *p* = 0.046). The mean MTV and TLG values were 35.74 ± 31.23 cm^3^ and 360.02 ± 427.84 in the CMR group and 62.49 ± 57.86 cm^3^ and 621.37 ± 566.54 in the N-CMR group, respectively*. *The frequencies of nodal involvement were also different between the CMR and N-CMR groups (27 of 57 patients, 47.36%, and 17 of 25 patients, 68.00%, respectively; *p* = 0.085). There were no significant differences in the mean SUVmax, SUVmean and age between the two groups (*p* = 0.292, *p* = 0.624, *p* = 0.778, respectively), nor in the frequencies of pathology findings, FIGO stage or lumboaortic nodal involvement (*p* = 0.936, *p* = 0.236 and *p* = 0.649, respectively).

**Table 2:** Comparison of continuous variables between the CMR and N-CMR groups (Student’s *t* test results)

Variable	Group		*p* value
	CMR	N-CMR	
Metabolic PET parameters			
SUVmax	16.57 ± 10.18	18.73 ± 7.58	0.292
SUVmean	9.27 ± 4.83	9.73 ± 3.33	0.626
MTV (cm^3^)	35.74 ± 31.23	62.49 ± 57.86	0.008
TLG	360.02 ± 427.84	621.37 ± 566.54	0.046
Clinical parameters			
Age at diagnosis (years)	56.8 ± 13.5	55.8 ± 16.1	0.763

The data presented are means ± SD

**Table 3:** Comparison of categorical variables between the CMR and N-CMR groups (Pearson’s chi-squared test results)

Variable	Group		*p* value
	CMR	N-CMR	
Pathology			
SCC	44 (53.7)	20 (24.4)	0.936
AC	11 (13.4)	4 (4.9)	
Non-SCC, non-AC	2 (2.4)	1 (1.2)	
FIGO stage			
IIA	1 (1.8)	2 (8.0)	0.236
IIB	37 (64.9)	17 (68.0)	
IIIA	4 (7.0)	0 (0.0)	
IIIB	10 (17.5)	2 (8.0)	
IVA	5 (8.8)	4 (16.0)	
Nodal status			
LN (any site)			
LN+	27 (47.4)	17 (68.0)	0.085
LN−	30 (52.6)	8 (32.0)	
LN (lumboaortic)			
LN+	7 (12.3)	4 (16.0)	0.649
LN−	50 (87.7)	21 (84.0)	

The data presented are number (%) of patients

*SCC* squamous cell carcinoma, *AC* adenocarcinoma, *LN* lymph nodes

### Logistic regression

All the variables were tested in a univariate logistic regression analysis to evaluate their relationship with the metabolic response to therapy (Table 4).

**Table 4:** Logistic regression analysis: associations with metabolic response to treatment

Independent variables	*p* value	Odds Ratio	95% CI	Nagelkerke *R*^2^
MTV	**0.019**	**1.015**	**1.002–1.028**	**0.110**
TLG	**0.045**	**1.001**	**1.000–1.002**	**0.081**
SUVmax	0.349	–	–	–
SUVmean	0.669	–	–	–
Age	0.759	–	–	–
FIGO stage	0.692	–	–	–
SCC	0.936	–	–	–
AC	0.729	–	–	–
Non-SCC, non-AC	0.939	–	–	–
LN involvement				
Any site	**0.088**	**2.361**	**0.879–6.343**	**0.051**
Lumboaortic	0.650	–	–	–

Variables with values in bold are independent predictors of response to therapy assuming significance at *p* < 0.1

*SCC* squamous cell carcinoma, *AC* adenocarcinoma, *LN* lymph nodes

Accepting *p* < 0.1 as the level of significance, the only independent predictors of response to therapy were MTV (*p* = 0.019, OR = 1.015, 95% CI = 1.002–1.028, Nagelkerke *R*^2^ = 0.110), TLG (*p* = 0.045, OR = 1.001, 95% CI = 1.000–1.002, Nagelkerke *R*^2^ = 0.081) and nodal involvement (PET-positive lymph nodes at any site; *p* = 0.088, OR = 2.361, 95% CI = 0.879–6.343, Nagelkerke *R*^2^ = 0.051). It was not possible to perform a multivariate logistic regression using both MTV and TLG as variables because, as expected, they were correlated (correlation test *p* < 0.0001, Pearson correlation = 0.786). MTV and nodal involvement were included in the multivariate backward logistic regression. However, the best logistic model was a model with only MTV as independent variable.

## Discussion

SUVmax is the most used PET parameter in the restaging of LACC patients. In our study, the EORTC classification was able to predict the OS in these patients, as demonstrated by Kaplan-Meier survival analysis. Patients with CMR after CCRT showed a better OS than patients with N-CMR (PMR, SMD, PMD). This observation could lead physicians to consider other adjuvant treatments in N-CMR patients (e.g. adjuvant chemotherapy) to improve prognosis and survival. Our findings are similar to those reported by Grigsby et al. [22]. In their study they evaluated 152 LACC patients who had undergone FDG PET/CT before and after treatment, : a CMR on PET was predictive of good survival (92% 5-year survival) while a PMR was predictive of poor survival (46% 5-year survival) and a PMD in 18 patients was predictive of death from cervical cancer in 17. These results indicating a good clinical outcome in LACC patients with a CMR to treatment as assessed by PET/CT were confirmed by Beriwal et al. [25] in a study in 155 patients.

We then sought to determine if some pretreatment PET parameters (SUVmax, SUVmean, MTV and TLG) and clinical parameters (age, pathology findings, FIGO stage and nodal involvement) could predict response to treatment. Although numerous published studies have investigated the prognostic value of FDG PET/CT in LACC, few of them assessed the role of volume-based and intensity-based PET parameters including MTV and TLG. In our cohort of patients, the statistical analysis indicated that pretreatment MTV and TLG and nodal involvement were predictors of response to therapy (MTV OR = 1.015, TLG OR = 1.001, nodal involvement OR = 2.361). Higher MTV and TLG values increase the risk of a non-complete metabolic response to CCRT. The presence of pretreatment PET-positive lymph nodes also increases the risk of a non-complete metabolic response to therapy. Pretreatment MTV was found to be the most significant predictor. However, the Nagelkerke *R*^2^ value in the univariate logistic regression analysis of the association between MTV and metabolic response was 0.110. This value indicates that the added value of MTV in predicting response to therapy is low. This could be due to the fact that there were relatively few patients in the N-CMR group (25 out 82). Further investigations including a larger number of patients are needed to assess the value of pretreatment MTV as a predictor of metabolic response to CCRT.

Liang et al. [37] found that pretreatment TLG was able to independently predict survival in patients with LACC. Ueno et al. [38] found that MTV and TLG of the primary tumour were significantly higher in nonresponders than in responders. In our study, SUVmax, SUVmean, age, pathology findings, FIGO stage and lumboaortic nodal involvement were not predictors of response to treatment. Although NCCN guidelines recommend that a restaging FDG PET/CT scan be performed 3–6 months after the end of CCRT, there is no complete agreement regarding the timing.

As in the largest studies in the literature, a limitation of our study was also the timing of the posttreatment PET/CT scan that ranged from 3 to 12 months (mean 6.7 months) after completion of treatment. Grisby et al. [22] performed restaging PET/CT at 1 to 12 months (mean 3 months) after treatment, and Onal et al. [39] performed FDG PET/CT at 3 to 10 months (median 4 months) after treatment. Another limitation of our study concerns patient classification: although patients were originally classified using the EORTC criteria into four groups (patients with CMR, PMR, SMD and PMD), we had to group those with PMR, SMD and PD together in the N-CMR group because of the small numbers of patients with PMR (eight) and SMD (two). A larger study, including more patients could better explore the benefit of grouping patients using the EORTC criteria.

### Conclusion

^18^F-FDG PET/CT-based response assessment using the EORTC criteria reliably predicts OS in LACC patients treated with CCRT. In our cohort of patients, pretreatment MTV and TLG and nodal involvement were predictors of response to treatment. MTV was the best predictor of response. However, its additional risk value seems to be low (MTV OR = 1.015). Further studies including a larger number of patients are needed to confirm our preliminary findings about the value of MTV, TLG and nodal involvement in response prediction.
